# Association Between Dietary Patterns and Dyslipidemia in Korean Women

**DOI:** 10.3389/fnut.2021.756257

**Published:** 2022-01-14

**Authors:** Jeonghee Lee, Tung Hoang, Seohyun Lee, Jeongseon Kim

**Affiliations:** Department of Cancer Biomedical Science, National Cancer Center Graduate School of Cancer Science and Policy, Goyang-si, South Korea

**Keywords:** dietary pattern, factor analysis, dyslipidemia, Korean women, menopausal status

## Abstract

**Background:**

The prevalence of dyslipidemia among Korean women differs significantly according to menopausal status. This study aimed to identify major dietary patterns among Korean women and examine their associations with the prevalence of dyslipidemia and its components.

**Methods:**

This study recruited 6,166 women from the Cancer Screenee Cohort 2007–2019 from the National Cancer Center of Korea. Dietary patterns were identified using factor analysis. Multivariable logistic regression was performed to calculate odds ratios (ORs) and 95% confidence intervals (CIs) for the associations between dietary patterns and the prevalence of dyslipidemia and its components, including hypercholesterolemia, hypertriglyceridemia, hypo-high-density lipoprotein (HDL) cholesterol, and hyper-low-density lipoprotein (LDL) cholesterol. Stratification analyses were performed for the premenopausal and postmenopausal subgroups.

**Results:**

The factor analysis identified three main dietary patterns, including traditional, western, and prudent dietary patterns. Compared with those with the lowest pattern scores, those with the highest pattern scores of the traditional (OR = 1.32, 95% CI = 1.05–1.67) and western (OR = 1.40, 95% CI = 1.11–1.78) diets had a higher prevalence of hyper-LDL cholesterol. When accounting for menopausal status in the analysis, traditional (OR = 1.44, 95% CI = 1.10–1.89) and western (OR = 1.43, 95% CI = 1.09–1.88) diets were still associated with hyper-LDL cholesterol in postmenopausal women. Additionally, consumption of a traditional diet was associated with a decreased prevalence of hypertriglyceridemia (OR = 0.73, 95% CI = 0.54–0.99), and consumption of a western diet was associated with an increased prevalence of hypercholesterolemia (OR = 1.41, 95% CI = 1.11–1.79) but a reduced prevalence of hypo-HDL cholesterol (OR = 0.60, 95% CI = 0.36–0.99). However, the prudent dietary pattern was not significantly associated with dyslipidemia and its components in the group of all women or the subgroups according to menopausal status.

**Conclusion:**

There were significant associations between the traditional and western dietary patterns and hyper-LDL cholesterol in the entire group and postmenopausal subgroup of women. In the perspective of energy restriction, our findings recommend women not to eat either traditional or western diets excessively or too frequently. Menopause may induce the effect of both the traditional diet on triglyceride reduction and the western diet on increasing total cholesterol.

## Introduction

Cardiovascular disease is the leading cause of non-communicable disease-related morbidity and mortality, accounting for an estimated 17.8 million deaths worldwide (1–3). It has been reported that dyslipidemia is the second greatest contributor to the risk of cardiovascular disease in Korea (4). Dyslipidemia is characterized by an abnormality in the lipid profile, including hypertriglyceridemia, hypercholesterolemia, hypo-high-density lipoprotein (HDL) cholesterol, and hyper-low-density lipoprotein (LDL) cholesterol. In this study, dyslipidemia was diagnosed when at least one of the above factors was present, the patient was prescribed medication for the treatment of dyslipidemia, or a previous diagnosis of dyslipidemia was established (5). Data from the Korean National Health and Nutrition Examination Survey (KNHANES) revealed that the prevalence of hypercholesterolemia increased consistently from 8.8% in 2007 to 18.0% in 2018, and the prevalence of dyslipidemia was very different between men and women, with rates of 45.6 and 31.3%, respectively (5). Notably, there was a rapid increase in the dyslipidemia prevalence in women older than 50–55 years (5). Postmenopausal women also had much higher serum concentrations of triglycerides, total cholesterol, and LDL cholesterol, and a remarkably lower concentration of HDL cholesterol than premenopausal women (4).

Aging has histologically contributed to lipid abnormalities through changes in the liver sinusoidal endothelium, lipoprotein disposition, and endocytosis and hepatic blood flow reductions (6). In addition, excessive adipocyte and adipose tissue accumulation can promote organellar dysfunction, hormone dysregulation, fatty acid storage impairment, and lipotoxicity to non-adipose tissue organs (7). Other lifestyle factors, such as smoking, drinking, and physical inactivity, may directly predispose individuals to dyslipidemia or indirectly affect through adipocity accumulation (7). Among genetic factors, hormonal abnormalities, and lifestyle factors, dietary intake plays an important role in the progression of dyslipidemia (8–10).

According to the Korean Society of Lipid and Atherosclerosis, the total daily energy intake in women has been constantly maintained over the past decade at 1,549 kcal in 2007 and 1,769 kcal in 2015 (5). However, the percentage of women with excessive energy intake increased from 10.0% in 2007 to 18.8% in 2015; this result can be explained by an increase in fat intake, which was 18.4% in 2007 and 21.8% in 2015 (5). We recently performed a systematic review and meta-analysis of the effects of single food items, nutrients, the dietary index, and dietary patterns on lipid profiles in the Korean population (11). However, dietary patterns that were identified from individual studies, such as the KNHANES and the industrialized community and rural area (Ansan and Ansung) cohorts, were mostly investigated in terms of the prevalence of elevated triglycerides and reduced HDL cholesterol (11). These outcomes were considered components of the metabolic syndrome, and the definition of lipid concentration was also different from the definition of dyslipidemia (11). The role of dietary intake on overall dyslipidemia as well as the components of total cholesterol and LDL cholesterol has not been widely investigated in the Korean population. Furthermore, hormonal changes during the menopausal transition period, including the increase in androgens and the decrease in circulating estrogen, are associated with the disorder of several serum lipids (12). Differences in the dysregulation of lipid metabolism between premenopausal and postmenopausal women may interact with the effect of dietary intake on dyslipidemia. Therefore, the current study aimed to identify the main dietary patterns and elucidate their associations with the prevalence of dyslipidemia and its components by comparing the highest with the lowest pattern scores in Korean women according to menopausal status.

## Materials and Methods

### Study Population

**Figure 1:**
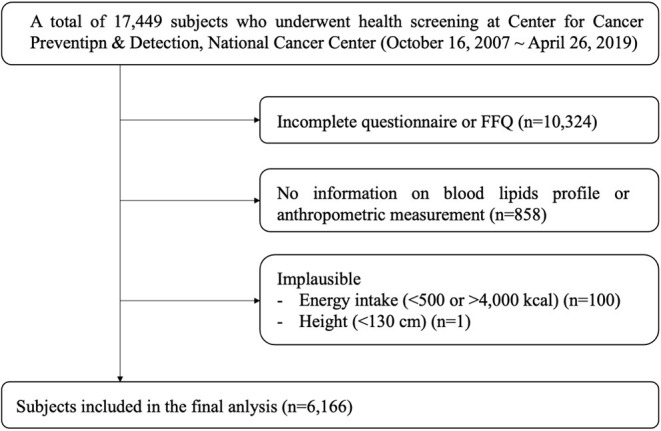
Flow chart of the participant selection process.

Data on 17,449 women who underwent health screening at the Center for Cancer Prevention and Detection, during 2007–2019 were obtained from the National Cancer Center, Korea (Figure 1). The study design is described elsewhere (13). Subjects participated in the study voluntarily, and the study protocol was approved by the National Cancer Center (number NCC2019-0204), Korea. After excluding women who did not complete the semi-quantitative food frequency questionnaire (semi-quantitative FFQ) or the general questionnaire (*n* = 10,324) and those who did not have blood lipid profile or anthropometric measurement data (*n* = 858), a total of 6,267 women subjects remained. We further excluded subjects with an implausible energy intake (<500 or >4,000 kcal, *n* = 100) or height (<130 cm, *n* = 1). Finally, we analyzed the data of the remaining 2,409 premenopausal and 3,757 postmenopausal women.

### Data Collection

All participants were asked to complete a self-administered questionnaire with questions related to their demographic and lifestyle data including age, educational level, household income, marital status, smoking status, alcohol consumption, regular exercise, and menopausal status.

Dietary intake was assessed by using the validated semi-quantitative FFQ for 106 food items (14). The average frequency of servings (never or rarely, once a month, 2–3 times a month, 1–2 times a week, 3 or 4 times a week, 5 or 6 times a week, once a day, twice a day, and 3 times a day) and the average portion size (small, medium, and large) were recorded to estimate the average weight of and energy intake from food items during the last year. For the semi-quantitative approach, small and large portion sizes were defined as 0.5 and 1.5 times of a standardized (medium) portion size, respectively. Pictures of the average portion size of each food item were presented to the participants. The daily intake according to weight (g/day) of 410 food components was calculated, and the foods were classified into 24 groups based on the nutrient profiles and culinary usage of each food item. These 24 groups included seasonings, vegetables, legumes and their products, potatoes and starches, fish and shellfish, seaweeds, mushrooms, kimchi, bread and snacks, meat and its products, noodles, pizza and hamburgers, poultry, carbonated beverages, rice cakes, whole grains, refined grains, fruits, milk and dairy products, seeds and nuts, eggs, coffee and tea, sugar and sweets, and oils and fats. Furthermore, daily energy and macronutrient consumption were estimated for all the women and menopause-specific subgroups.

### Anthropometric and Biochemical Parameters

Height was measured to the nearest 0.1 cm, and weight was measured to the nearest 0.1 kg while the subjects wore light clothes, without shoes, using automatic height and weight measurements (DS-102, Dong Sahn Jenix Co., Ltd., Seoul, Korea). Body mass index (BMI) was defined as the weight (kg) divided by the square of the height (m). Waist circumference was measured at the umbilical level using a measuring tape. Blood pressure was measured with the participants in the seated position after 15 min of rest, using an automatic blood pressure monitor (HBP-9020, Omron, Kyoto, Japan).

Venous blood samples for the biochemical analyses were collected from participants by venipuncture following 8 h of fasting. Triglycerides, HDL cholesterol, total cholesterol (Kyowa Medex, Tokyo, Japan), and fasting glucose (Denka Seiken, Tokyo, Japan) were determined using a chemical analyzer (TBA-200FR, Toshiba, Tokyo, Japan). LDL cholesterol was estimated using Friedwald equation when TG was <400 mg/dL. Dyslipidemia was defined as the presence of at least one of the following criteria: triglycerides ≥200 mg/dL, total cholesterol ≥240 mg/dL, HDL cholesterol <40 mg/dL, LDL cholesterol ≥160 mg/dL, previous diagnosis of dyslipidemia, and use of lipid-lowering drugs.

### Statistical Analysis

The dietary patterns were identified by factor analysis using the FACTOR procedure. An eigenvalue >1.5 was used to determine whether a factor should be considered as a major dietary pattern. Varimax rotation was applied to review the correlations between variables and factors. Food groups with either positive or negative loadings in each pattern indicated the direct or inverse relationships with that pattern. For each subject, the factor scores for each dietary pattern were calculated by summing the intake of each food group, weighted by the factor loading. The derived patterns were named based on the most positively loaded foods associated with the patterns. Participants had their own factor score for identified patterns, and they were categorized into tertiles for every emerged pattern by the subject's factor score.

Differences in the distribution of covariates across tertiles of each dietary pattern score were examined using the chi-square test for categorical variables and a generalized linear model for continuous variables. Multivariable-adjusted logistic regression analysis was performed to compare the odds ratios (ORs) and 95% confidence intervals (CIs) for dyslipidemia among tertiles of each dietary pattern score. The logistic regression model was adjusted for age, education, household income, smoking status, alcohol consumption, regular exercise, BMI, and total energy intake. P for trend was calculated by assigning the median value of each tertile category as a continuous variable to examine the linear trend of dyslipidemia according to the tertiles of the three dietary patterns. Subgroup analysis considering menopausal status was also performed.

Nationwide studies in Korea showed that the prevalence of dyslipidemia 2010–2012, hypercholesterolemia 2007–2016, elevated triglycerides 2008–2013, and reduced HDL cholesterol 2008–2013 were 14.9–52.9% (15), 9.3–20.2% (15), 28.2–29.4% (16), and 47.0–54.2% (16), respectively. Additionally, the ORs for the measurement of dietary patterns in relation to reduced HDL cholesterol and elevated triglycerides ranged from 0.53 to 1.53, and the control-to-case ratio was assumed to be 7.76 (592 cases of metabolic syndrome vs. 4,597 controls with non-metabolic syndrome) according to a previous study (17). Given the statistical power of 80%, the two-sided significance level of 0.05, and the dropout rate of 10%, the maximum number of required participants was 5,259 (18). Thus, the current study had adequate power to detect the magnitude of associations. All statistical analyses were performed using SAS software (version 9.4, SAS Institute, Inc., Cary, NC, USA) and a *P*-value of 0.05 was considered to be statistically significant.

## Results

Table 1 shows the general characteristics of the study participants according to dyslipidemia status. Women with dyslipidemia were higher age, BMI, blood pressure, fasting glucose, triglyceride, and total and LDL cholesterol levels, as well as a higher prevalence of postmenopausal status, than those without dyslipidemia (*p* < 0.001). In contrast, a higher education level, household income, and alcohol consumption and more regular exercise were observed in women without dyslipidemia (*p* < 0.001).

**Table 1:** General characteristics according to dyslipidemia status.

**Variable**	**Total** **(*n* = 6,166)**	**Dyslipidemia** **(*n* = 2,045)**	**Non-dyslipidemia** **(*n* = 4,121)**	***P-*value**
General information				
Age (years)	52.1 ± 8.1	55.4 ± 7.3	50.4 ± 7.9	<0.001
Education level				
Less than middle school	872 (14.1)	364 (17.8)	508 (12.3)	<0.001
High school	2,408 (39.1)	824 (40.3)	1,584 (38.4)	
College or more	2,539 (41.2)	717 (35.1)	1,822 (44.2)	
Missing	347 (5.6)	140 (6.9)	207 (5.0)	
Household income (10,000 won/month)				
<200	1,204 (19.5)	473 (23.1)	731 (17.7)	<0.001
200–400	2,030 (32.9)	692 (33.8)	1,338 (32.5)	
>400	2,096 (34.0)	551 (26.9)	1,545 (37.5)	
Missing	836 (13.6)	329 (26.9)	507 (12.3)	
Marital status				
With spouse	4,953 (80.3)	1,639 (80.2)	3,312 (70.4)	0.88
Without spouse	1,110 (18.0)	370 (18.1)	740 (18.0)	
Missing	103 (1.7)	36 (1.8)	67 (1.6)	
Smoking status				
Non-smoker	5,652 (91.7)	1,865 (91.2)	3,787 (91.9)	0.18
Ex-smoker	246 (4.0)	72 (3.5)	174 (4.2)	
Current smoker	177 (2.9)	67 (3.3)	110 (2.7)	
Missing	91 (1.5)	41 (2.0)	50 (1.2)	
Alcohol consumption				
No (0 g/d)	3,558 (57.7)	1,312 (64.2)	2,246 (54.5)	<0.001
Light drinkers (<2.4 g/d)	1,230 (19.9)	355 (17.4)	875 (21.2)	
Heavy drinkers (≥2.4 g/d)	1,209 (19.9)	314 (15.4)	895 (21.7)	
Missing	169 (2.7)	64 (3.1)	105 (2.6)	
Regular exercise				
No	2,704 (43.9)	885 (43.3)	1,819 (44.1)	0.47
Yes	3,344 (54.2)	1,124 (55.0)	2,220 (53.9)	
Missing	118 (1.9)	36 (1.8)	82 (2.0)	
Menopause status				
Premenopausal	2,409 (39.1)	419 (20.5)	1,990 (48.3)	<0.001
Postmenopausal	3,757 (60.9)	1,626 (79.5)	2,131 (51.7)	
Diabetics				
No	5,891 (95.5)	1,866 (91.3)	4,025 (97.7)	<0.001
Yes	275 (4.5)	179 (8.7)	96 (2.3)	
Hypertension				
No	4,718 (76.5)	1,350 (66.0)	3,368 (81.7)	<0.001
Yes	4,188 (67.9)	695 (34.0)	753 (18.3)	
Body mass index (kg/m^2^)	23.2 ± 3.0	24.1 ± 3.1	22.8 ± 2.9	<0.001
Systolic blood pressure (mmHg)	123.6 ± 14.9	126.9 ± 15.1	121.9 ± 14.4	<0.001
Diastolic blood pressure (mmHg)	74.2 ± 10.3	76.2 ± 10.2	73.2 ± 10.1	<0.001
Waist circumference (cm)	74.2 ± 10.3	76.6 ± 7.6	73.0 ± 7.2	<0.001
Fasting glucose (mg/dL)	91.3 ± 15.3	95.2 ± 19.2	89.3 ± 12.6	<0.001
Blood lipid profiles				
Triglyceride (mg/dL)	105.2 ± 63.9	143.2 ± 85.6	86.4 ± 37.4	<0.001
Total cholesterol (mg/dL)	201.9 ± 36.4	223.2 ± 43.9	191.4 ± 26.2	<0.001
HDL cholesterol (mg/dL)	62.6 ± 14.4	59.7 ± 16.2	64.1 ± 13.3	<0.001
LDL cholesterol (mg/dL)	118.3 ± 33.0	134.9 ± 41.5	110.1 ± 23.9	<0.001

*Categorical variables are summarized as numbers (percentages) and compared using the chi-square test. Continuous variables are summarized as mean±standard deviation and compared using the generalized linear model. LDL, low-density lipoprotein; HDL, high-density lipoprotein*.

Daily energy intake was not different between the premenopausal and postmenopausal women (Table 2). Compared with premenopausal women, postmenopausal women consumed more plant protein, carbohydrates, and fiber, but consumed less animal protein, plant fat, and animal fat (*p* < 0.001).

**Table 2:** Nutrient intake in Korean women.

**Variable**	**Total** **(*n* = 6,166)**	**Premenopausal** **(*n* = 2,045)**	**Postmenopausal** **(*n* = 4,121)**	***P-*value**
Energy (kcal/day)	1,610.4 (568.5)	1,624.9 (577.7)	1,601.1 (562.4)	0.11
Plant protein				
g/day	38.5 (6.3)	37.5 (5.9)	39.1 (6.4)	<0.001
% energy	9.3 (1.5)	9.1 (1.4)	9.5 (1.6)	<0.001
Animal protein				
g/day	24.0 (11.2)	25.1 (10.8)	23.3 (11.4)	<0.001
% energy	5.7 (2.7)	6.0 (2.6)	5.5 (2.7)	<0.001
Plant fat				
g/day	14.3 (6.2)	14.7 (6.1)	14.0 (6.3)	<0.001
% energy	7.6 (3.3)	7.9 (3.3)	7.5 (3.4)	<0.001
Animal fat				
g/day	16.1 (8.7)	17.7 (8.8)	15.1 (8.6)	<0.001
% energy	8.6 (4.6)	9.5 (4.7)	8.1 (4.5)	<0.001
Carbohydrate				
g/day	307.7 (35.3)	302.0 (34.4)	311.3 (35.4)	<0.001
% energy	74.4 (8.6)	73.0 (8.3)	75.3 (8.6)	<0.001

*Data are summarized as mean ± standard deviation and compared using the generalized linear model*.

The factor loading matrix of the factor analysis for the 24 food groups is presented in Table 3. As a result, three dietary patterns were derived by factor analysis, and the total proportion of the variance explained by the extracted dietary patterns was 30.13%. The traditional dietary pattern was characterized by a high intake of seasonings, vegetables, legumes, potatoes and starches, fish and shellfish, seaweeds, mushrooms, and kimchi and a low intake of refined grains. The western dietary pattern was characterized by a high consumption of bread and snacks, meats, noodles, pizza and hamburgers, carbonated beverages, eggs, sugar and sweets, and oils and fats and a low consumption of grains. The prudent dietary pattern was characterized by a high intake of vegetables, fish and shellfish, fruits, milk and dairy products, seeds and nuts, and eggs and a low intake of noodles, refined grains, sugar and sweets, and oils and fats.

**Table 3:** Factor loading matrix for three dietary patterns identified by factor analysis.

**Food groups**	**Traditional** **(*n =* 6,166)**	**Western** **(*n =* 6,166)**	**Prudent** **(*n =* 6,166)**
Seasonings	0.73		
Vegetables	0.68		0.31
Legumes and their products	0.59		
Potatoes and starches	0.57		
Fish and shellfish	0.52		0.21
Seaweeds	0.48		
Mushrooms	0.44		
Kimchi	0.32		
Bread and snacks		0.52	
Meat and its products		0.52	
Noodles		0.47	−0.22
Pizza and hamburgers		0.45	
Poultry		0.40	
Carbonated beverages		0.25	
Rice cakes			
Whole grains		−0.40	
Refined grains	−0.45	−0.67	−0.50
Fruits			0.57
Milk and dairy products			0.48
Seeds and nuts			0.39
Eggs		0.24	0.27
Coffee and tea			
Sugar and sweets		0.43	−0.48
Oils and fats	0.25	0.54	−0.55
Proportion of variance explained (%)	13.58	9.65	6.90

*Factor loading scores from −0.20 to 0.20 are not shown*.

Table 4 shows the general characteristics of the study participants according to the scores of the three dietary patterns. While subjects with higher scores who exhibited the traditional and prudent dietary patterns were more likely to be older, those with higher scores in the western dietary pattern were more likely to be younger (*p* < 0.001). Household income, tobacco smoking, alcohol consumption, and regular exercise were unequally distributed among the tertiles of all the dietary patterns. Additionally, the three dietary pattern scores were positively associated with total energy intake (*p* < 0.001). Regarding the lipid profile, while the average total cholesterol (*p* = 0.013) and LDL cholesterol (*p* = 0.012) concentrations were not equal among the tertiles of the traditional dietary pattern score, those of serum triglycerides (*p* < 0.001) and HDL cholesterol (*p* < 0.001) were significantly different between the tertiles of the western dietary pattern score. We observed unequal levels of all the components of the lipid profile between the tertiles of the prudent dietary pattern score (*p* ≤ 0.001).

**Table 4:** General characteristics by tertiles of dietary pattern score tertiles.

**Variables**	**Traditional (*n =* 6,166)**				**Western (*n =* 6,166)**				**Prudent (*n =* 6,166)**			
	**T1**	**T2**	**T3**	***P-*value**	**T1**	**T2**	**T3**	***P-*value**	**T1**	**T2**	**T3**	***P-*value**
**General information**												
Number of participants	2,056 (33.3)	2,055 (33.3)	2,055 (33.3)		2,056 (33.3)	2,055 (33.3)	2,055 (33.3)		2,056 (33.3)	2,055 (33.3)	2,055 (33.3)	
Age (years)	50.7 ± 8.0	51.7 ± 8.0	53.8 ± 7.9	<0.001	54.7 ± 7.6	51.8 ± 7.9	49.7 ± 7.9	<0.001	51.4 ± 8.4	52.1 ± 8.0	52.7 ± 7.8	<0.001
**Education level**												
Less than middle school	265 (12.9)	268 (13.0)	339 (16.5)	<0.001	435 (21.2)	241 (11.7)	196 (9.5)	<0.001	379 (18.4)	275 (13.4)	218 (10.6)	<0.001
High school	775 (37.7)	803 (39.1)	830 (40.4)		861 (41.9)	816 (39.7)	731 (35.6)		847 (41.2)	813 (39.6)	748 (36.4)	
College or more	872 (42.2)	885 (43.1)	782 (38.1)		593 (28.8)	902 (43.9)	1,044 (50.8)		700 (34.1)	856 (41.7)	983 (47.8)	
Missing	144 (7.0)	99 (4.8)	104 (5.1)		167 (8.1)	96 (4.7)	84 (4.1)		130 (6.3)	111 (5.4)	106 (5.2)	
**Household income (10,000 won/month)**												
<200	394 (19.2)	373 (18.2)	437 (21.3)	0.026	491 (23.9)	367 (17.9)	346 (16.8)	<0.001	468 (22.8)	402 (19.6)	334 (16.3)	<0.001
200–400	650 (31.6)	699 (34.0)	681 (33.1)		675 (32.8)	701 (34.1)	654 (31.8)		684 (33.3)	673 (32.8)	673 (32.8)	
>400	723 (35.2)	718 (34.9)	655 (31.9)		540 (26.3)	730 (35.5)	826 (40.2)		590 (28.7)	709 (34.5)	797 (38.8)	
Missing	289 (14.1)	265 (12.9)	282 (13.7)		350 (17.0)	257 (12.5)	229 (11.1)		314 (15.3)	271 (13.2)	251 (12.2)	
**Marital status**												
With spouse	1,627 (79.1)	1,667 (81.1)	1,659 (80.7)	0.372	1,654 (80.5)	1,650 (80.3)	1,649 (80.2)	0.759	1,642 (79.9)	1,653 (80.4)	1,658 (80.7)	0.895
Without spouse	389 (18.9)	360 (17.5)	361 (17.6)		358 (17.4)	374 (18.2)	378 (18.4)		376 (18.3)	365 (17.8)	369 (18.0)	
Missing	40 (2.0)	28 (1.4)	35 (1.7)		44 (2.1)	31 (1.5)	28 (1.4)		38 (1.9)	37 (1.8)	28 (1.4)	
**Smoking status**												
Non-smoker	1,866 (90.8)	1,875 (91.2)	1,911 (93.0)	0.002	1,931 (93.9)	1,912 (93.0)	1,809 (88.0)	<0.001	1,854 (90.2)	1,880 (91.5)	1,918 (93.3)	<0.001
Ex-smoker	99 (4.8)	93 (4.5)	54 (2.6)		62 (3.0)	70 (3.4)	114 (5.6)		92 (4.5)	87 (4.2)	67 (3.3)	
Current smoker	53 (2.6)	66 (3.2)	58 (2.5)		32 (1.6)	47 (2.3)	98 (4.8)		83 (4.0)	58 (2.8)	36 (1.8)	
Missing	38 (1.9)	21 (1.0)	32 (2.8)		31 (1.5)	26 (1.3)	34 (1.7)		27 (1.3)	30 (1.5)	34 (1.7)	
**Alcohol consumption**												
No (0 g/d)	1,146 (55.7)	1,141 (55.5)	1,271 (61.9)	<0.001	1,398 (68.0)	1,201 (58.4)	959 (46.7)	<0.001	1,150 (55.9)	1,156 (56.3)	1,252 (60.9)	0.002
Light drinkers (<2.4 g/d)	426 (20.7)	437 (21.3)	367 (17.9)		316 (15.4)	415 (20.2)	499 (24.3)		415 (20.2)	416 (20.2)	399 (19.4)	
Heavy drinkers (≥2.4 g/d)	419 (20.4)	429 (20.9)	361 (17.6)		290 (14.1)	382 (18.6)	537 (26.1)		436 (21.2)	425 (20.7)	348 (16.9)	
Missing	65 (3.2)	48 (2.3)	56 (2.7)		52 (2.5)	57 (2.8)	60 (2.9)		55 (2.7)	58 (2.8)	56 (2.7)	
**Regular exercise**												
No	1,012 (49.2)	895 (43.6)	797 (38.8)	<0.001	862 (41.9)	826 (40.2)	1,016 (49.4)	<0.001	1,110 (54.0)	887 (43.2)	707 (34.4)	<0.001
Yes	1,000 (47.6)	1,121 (54.6)	1,223 (59.5)		1,154 (56.1)	1,195 (58.2)	995 (48.4)		892 (43.4)	1,131 (55.0)	1,321 (64.3)	
Missing	44 (2.1)	39 (1.9)	35 (1.7)		40 (2.0)	34 (1.7)	44 (2.1)		54 (2.6)	37 (1.8)	27 (1.3)	
**Anthropometric and biochemical parameter**												
Body mass index (kg/m^2^)	23.0 ± 3.0	23.2 ± 3.0	23.4 ± 3.0	0.001	23.3 ± 3.0	23.2 ± 2.9	23.1 ± 3.0	0.107	23.4 ± 3.0	23.2 ± 3.1	23.0 ± 2.9	<0.001
**General information**												
Systolic blood pressure (mmHg)	122.5 ± 14.7	123.7 ± 15.2	124.5 ± 14.5	<0.001	125.1 ± 15.3	124.3 ± 14.8	121.3 ± 14.2	<0.001	124.2 ± 14.9	123.7 ± 14.9	122.8 ± 14.7	0.007
Diastolic blood pressure (mmHg)	73.5 ± 10.3	74.0 ± 10.5	74.9 ± 10.0	<0.001	74.9 ± 10.4	74.6 ± 10.3	72.9 ± 10.0	<0.001	74.7 ± 10.4	74.1 ± 10.3	73.7 ± 10.0	0.006
Waist circumference (cm)	73.7 ± 7.4	74.0 ± 7.4	74.8 ± 7.7	<0.001	74.7 ± 7.7	74.1 ± 7.4	73.7 ± 7.4	<0.001	74.7 ± 7.5	74.3 ± 7.6	73.5 ± 7.4	<0.001
Fasting glucose (mg/dL)	91.0 ± 15.5	90.9 ± 14.1	92.0 ± 16.3	0.028	93.0 ± 18.0	91.2 ± 14.3	89.7 ± 13.1	<0.001	91.3 ± 16.0	91.8 ± 15.5	90.8 ± 14.5	0.095
Energy (kcal)	1,540.9 ± 529.5	1,622.7 ± 535.6	1,667.6 ± 628.0	<0.001	1,535.4 ± 484.5	1,629.9 ± 545.0	1,665.9 ± 655.0	<0.001	1,527.7 ± 512.1	1,636.8 ± 547.5	1,666.7 ± 630.3	<0.001
**Blood lipid profiles**												
Triglyceride (mg/dL)	105.6 ± 67.8	103.0 ± 62.5	107.0 ± 61.1	0.129	111.0 ± 65.4	103.6 ± 63.0	101.1 ± 62.7	<0.001	110.7 ± 70.1	105.0 ± 61.1	100.0 ± 59.4	<0.001
Total cholesterol (mg/dL)	201.7 ± 36.3	200.4 ± 35.8	203.7 ± 36.9	0.013	201.4 ± 36.0	201.3 ± 36.7	203.1 ± 36.4	0.220	199.5 ± 36.1	201.7 ± 36.8	204.6 ± 36.1	<0.001
HDL cholesterol (mg/dL)	63.0 ± 14.7	62.5 ± 14.1	62.3 ± 14.6	0.220	61.3 ± 14.2	62.7 ± 14.4	63.9 ± 14.6	<0.001	61.0 ± 14.0	62.4 ± 14.5	64.4 ± 14.5	<0.001
LDL cholesterol (mg/dL)	117.6 ± 32.8	117.2 ± 32.6	120.0 ± 33.5	0.012	117.9 ± 32.6	117.9 ± 33.3	119.0 ± 33.0	0.478	116.4 ± 32.8	118.3 ± 33.1	120.2 ± 32.9	0.001

*Categorical variables are summarized as numbers (percentages) and compared using Chi-square test. Continuous variables are summarized as mean±standard deviation and compared using the generalized linear model. LDL, low-density lipoprotein; HDL, high-density lipoprotein*.

The association between dietary patterns and dyslipidemia and its components in Korean women is presented in Table 5. Compared with the lowest tertiles of the scores of the three dietary patterns, the highest tertiles were not associated with the prevalence of dyslipidemia. Regarding the components of dyslipidemia, the traditional dietary pattern was related to the prevalence of hyper-LDL cholesterol (OR = 1.32, 95% CI = 1.05–1.67). The highest tertile of the western dietary pattern was positively associated with hyper-LDL cholesterol (OR = 1.40, 95% CI = 1.10–1.78, p-trend = 0.01).

**Table 5:** Odds ratios and 95% confidence intervals for the association between tertiles of dietary pattern score and the dyslipidemia and its components in women.

	**Tertiles of dietary pattern score**			
	**1st tertile (*n =* 2,056)**	**2nd tertile (*n =* 2,055)**	**3rd tertile (*n =* 2,055)**	*P* for trend^*^
Traditional				
Dyslipidemia	1.00	0.85 (0.71–1.01)	0.91 (0.76–1.08)	0.35
Triglyceride (≥200 mg/dL)	1.00	0.77 (0.59–1.01)	0.78 (0.60–1.02)	0.09
Total cholesterol (≥240 mg/dL)	1.00	0.90 (0.74–1.10)	1.02 (0.84–1.24)	0.73
HDL-cholesterol (<40 mg/dL)	1.00	0.89 (0.61–1.28)	0.88 (0.61–1.28)	0.54
LDL–cholesterol (≥160 mg/dL)	1.00	1.07 (0.84–1.36)	1.32 (1.05–1.67)	0.01
Western				
Dyslipidemia	1.00	0.97 (0.81–1.15)	1.10 (0.92–1.31)	0.28
Triglyceride (≥200 mg/dL)	1.00	0.83 (0.64–1.08)	0.75 (0.57–1.00)	0.05
Total cholesterol (≥240 mg/dL)	1.00	1.19 (0.97–1.45)	1.38 (1.12–1.69)	0.003
HDL–cholesterol (<40 mg/dL)	1.00	0.74 (0.51–1.06)	0.77 (0.53–1.13)	0.19
LDL–cholesterol (≥160 mg/dL)	1.00	1.24 (0.98–1.57)	1.40 (1.10–1.78)	0.01
Prudent				
Dyslipidemia	1.00	0.97 (0.82–1.16)	1.02 (0.85–1.22)	0.81
Triglyceride (≥200 mg/dL)	1.00	0.91 (0.70–1.19)	0.82 (0.62–1.08)	0.15
Total cholesterol (≥240 mg/dL)	1.00	1.03 (0.84–1.26)	1.13 (0.92–1.38)	0.25
HDL–cholesterol (<40 mg/dL)	1.00	0.84 (0.59–1.19)	0.68 (0.47–1.00)	0.05
LDL–cholesterol (≥160 mg/dL)	1.00	1.03 (0.81–1.31)	1.22 (0.96–1.55)	0.09

*LDL, low density lipoprotein; HDL, high-density lipoprotein.*

* *Adjusted for age, education, household income, smoking status, alcohol consumption, regular exercise, body mass index, total energy intake, and antihyperlipidemia and antidiabetes medications. P for trend was calculated using the median value of each tertile category as a continuous variable*.

Table 6 presents the associations of dietary patterns and menopausal status with the prevalence of dyslipidemia and its components. In the premenopausal women, none of the dietary patterns were associated with dyslipidemia or its components. In postmenopausal women, the traditional pattern was inversely associated with the prevalence of hypertriglyceridemia (OR = 0.73, 95% CI = 0.54–0.99), but positively associated with hyper-LDL cholesterol (OR = 1.44, 95% CI = 1.10–1.89). Furthermore, the western pattern was inversely associated with the prevalence of hypo-HDL cholesterol (OR = 0.60, 95% CI = 0.36–0.99) but positively associated with hypercholesterolemia (OR = 1.41, 95% CI = 1.11–1.79) and hyper-LDL cholesterol (OR = 1.43, 95% CI = 1.09–1.88).

**Table 6:** Odds ratios and 95% confidence intervals for the association between tertiles of dietary pattern score and the dyslipidemia and its components by menopausal status.

	**Tertiles of dietary pattern score**			
	**1st tertile**	**2nd tertile**	**3rd tertile**	*P* for trend^*^
**Premenopausal Women (*n =* 2,409)**				
Traditional				
Number of participants	803	803	803	
Dyslipidemia	1.00	0.85 (0.63–1.15)	0.95 (0.69–1.31)	0.75
Triglyceride (≥200 mg/dL)	1.00	0.98 (0.59–1.61)	0.89 (0.51–1.53)	0.70
Total cholesterol (≥240 mg/dL)	1.00	0.83 (0.57–1.23)	0.80 (0.52–1.21)	0.29
HDL–cholesterol (<40 mg/dL)	1.00	1.02 (0.56–1.86)	1.20 (0.64–2.28)	0.57
LDL–cholesterol (≥160 mg/dL)	1.00	0.69 (0.42–1.14)	1.10 (0.67–1.80)	0.67
Western				
Number of participants	803	803	803	
Dyslipidemia	1.00	0.79 (0.55–1.14)	1.14 (0.81–1.60)	0.16
Triglyceride (≥200 mg/dL)	1.00	0.48 (0.26–0.88)	0.71 (0.42–1.20)	0.48
Total cholesterol (≥240 mg/dL)	1.00	1.18 (0.72–1.94)	1.58 (1.00–2.49)	0.03
HDL–cholesterol (<40 mg/dL)	1.00	0.71 (0.34–1.47)	0.95 (0.49–1.85)	0.86
LDL–cholesterol (≥160 mg/dL)	1.00	1.12 (0.60–2.07)	1.48 (0.84–2.61)	0.12
Prudent				
Number of participants	803	803	803	
Dyslipidemia	1.00	1.04 (0.77–1.41)	1.10 (0.80–1.53)	0.56
Triglyceride (≥200 mg/dL)	1.00	1.13 (0.69–1.84)	0.93 (0.53–1.63)	0.85
Total cholesterol (≥240 mg/dL)	1.00	1.20 (0.80–1.80)	1.49 (0.98–2.26)	0.06
HDL–cholesterol (<40 mg/dL)	1.00	0.78 (0.43–1.42)	0.82 (0.43–1.55)	0.50
LDL–cholesterol (≥160 mg/dL)	1.00	1.36 (0.83–2.20)	1.29 (0.76–2.20)	0.33
**Postmenopausal Women (*****n** **=*** **3,757)**			
Traditional				
Number of participants	1,253	1,252	1,252	
Dyslipidemia	1.00	0.87 (0.70–1.08)	0.90 (0.73–1.11)	0.04
Triglyceride (≥200 mg/dL)	1.00	0.71 (0.52–0.98)	0.73 (0.54–0.99)	0.07
Total cholesterol (≥240 mg/dL)	1.00	0.96 (0.75–1.22)	1.10 (0.87–1.38)	0.35
HDL–cholesterol (<40 mg/dL)	1.00	0.82 (0.51–1.30)	0.77 (0.49–1.21)	0.29
LDL–cholesterol (≥160 mg/dL)	1.00	1.27 (0.96–1.69)	1.44 (1.10–1.89)	0.01
Western				
Number of participants	1,253	1,252	1,252	
Dyslipidemia	1.00	1.07 (0.88–1.31)	1.10 (0.88–1.37)	0.39
Triglyceride (≥200 mg/dL)	1.00	1.00 (0.75–1.34)	0.73 (0.52–1.03)	0.09
Total cholesterol (≥240 mg/dL)	1.00	1.24 (0.99–1.55)	1.41 (1.11–1.79)	0.01
HDL–cholesterol (<40 mg/dL)	1.00	0.77 (0.50–1.17)	0.60 (0.36–0.99)	0.04
LDL–cholesterol (≥160 mg/dL)	1.00	1.31 (1.01–1.69)	1.43 (1.09–1.88)	0.01
Prudent				
Number of participants	1,253	1,252	1,252	
Dyslipidemia	1.00	0.89 (0.72–1.11)	0.93 (0.75–1.15)	0.53
Triglyceride (≥200 mg/dL)	1.00	0.82 (0.60–1.12)	0.74 (0.54–1.02)	0.07
Total cholesterol (≥240 mg/dL)	1.00	0.93 (0.74–1.18)	0.95 (0.75–1.20)	0.68
HDL–cholesterol (<40 mg/dL)	1.00	0.88 (0.57–1.38)	0.64 (0.40–1.03)	0.07
LDL–cholesterol (≥160 mg/dL)	1.00	0.91 (0.69–1.20)	1.11 (0.85–1.45)	0.36

*LDL, low density lipoprotein; HDL, high-density lipoprotein.*

* *Adjusted for age, education, household income, smoking status, alcohol consumption, regular exercise, body mass index, total energy intake, and antihyperlipidemia and antidiabetes medications. P for trend was calculated using the median value of each tertile category as a continuous variable*.

## Discussion

In this cross-sectional study, factor analysis revealed three main dietary patterns in Korean women, namely, the traditional, western, and prudent dietary patterns. Overall, the prudent dietary pattern was not associated with dyslipidemia or its components. The high consumption of food groups associated with the traditional and western patterns was positively associated with the prevalence of hyper-LDL cholesterol. In postmenopausal women, there were additional significant associations of the high consumption of food groups in the traditional dietary pattern with the low prevalence of hypertriglyceridemia, as well as of the western dietary pattern with the high prevalence of hypercholesterolemia and the low prevalence of hypo-HDL cholesterol in Korean postmenopausal women.

The traditional dietary pattern included kimchi, seafood, and vegetables. In our previous report of the Cancer Screenee Cohort 2002–2007, a traditional dietary pattern was not associated with metabolic syndrome (OR = 1.05, 95% CI = 0.79–1.40) or elevated triglycerides (≥150 mg/dL) (OR = 1.03, 95% CI = 0.79–1.34) in Korean women (19). Although there was a positive association between the high consumption of food groups in the traditional dietary pattern group and the prevalence of low HDL cholesterol (<50 mg/dL), the difference was only moderately significant (OR = 1.22, 95% CI = 1.01–1.46) (19). A nonsignificant association between the Korean traditional dietary pattern was also reported in the Cancer Screening Cohort 2007–2014, with ORs (95% CIs) for elevated triglycerides and low HDL cholesterol of 0.88 (0.70–1.11) and 1.13 (0.90–1.41), respectively (17). Although the criteria for elevated LDL cholesterol (≥130 mg/dL) in metabolic syndrome are different from those of hyper-LDL cholesterol in dyslipidemia, the conventional Korean dietary pattern was not associated with the prevalence of elevated LDL cholesterol (OR = 1.09, 95% CI = 0.82–1.44) in 5,837 women from the KNHANES 2007–2009 (20). The nutrition guidelines in Western countries generally recommend diets high in vegetables, fruits, and fish (21). Adherence to a Mediterranean diet improved of lipid profile, with a lower odds of optimal HDL cholesterol (22). In a randomized controlled trial, participants who followed a Dietary Approaches to Stop Hypertension (DASH) diet exhibited a significant reduction in LDL and HDL cholesterol, apolipoprotein A-I, and intermediate-density lipoprotein and large LDL particles (23). Several nutrients in fruits and vegetables, such as fiber, vitamins, and polyphenols were reported to be beneficial to health outcomes (24). Additionally, the lipid-lowering mechanism of kimchi and its ingredients such as β-sitosterol in Chinese cabbage, *S*-methlycysteinsulfoxide and *S*-allylcysteinsulfoxide in garlic, and capsaicin in red pepper, was reported (25). These bioactive compounds can inhibit the absorption and synthesis of cholesterol and induce the secretion of serum cholesterol into extracirculation as bile. However, Asian populations, especially elderly Korean, consume a relatively high amount of carbohydrates (>70% of total energy intake) (24). Excessive consumption of carbohydrates in the traditional diet can thus increase triglycerides and decrease HDL cholesterol by inhibiting lipoprotein lipase action through apolipoprotein CIII (26).

The western dietary pattern included large amounts of oils and fats, meat, and fast food and small amounts of grains. In the current study, we found that the consumption of food groups by people with in the western dietary pattern was positively associated with the prevalence of hyper-LDL cholesterol. This was consistent with findings from a study in Chinese women (*N* = 2,468), which showed high levels of LDL cholesterol among those with a high score in the western dietary pattern (27). Similar findings were also observed in the 2012 National Health and Nutrition Survey in Japan (*N* = 6,679), with ORs (95% CIs) for the association between the western dietary pattern and elevated LDL cholesterol (≥140 mg/dL) of 1.75 (1.30–2.35) (28). In the human body, most of the fatty acids can be synthesized from carbohydrate, fat, and protein intake (12). While omega-3 fatty acids show anti-inflammatory, cardioprotective, and insulin-sensitive effects, omega-6 fatty acids exhibit proinflammatory effects, and increase the risk of cardiometabolic disorders (12). In the meantime, western diets are inadequate in omega-3 fatty acids and contain excessive amounts of omega-6 fatty acids (12). Additionally, undesirable effects of saturated fatty acids and heme iron in red and processed meat can reduce LDL-receptor-mediated clearance and act as a catalyst of oxidative damage (29).

The prudent dietary pattern was characterized by the high intake of fruits and milk and dairy products and the low intake of oils, sweets, and refined grains. The potentially protective effect of fruits or milk and dairy products on lipid profiles in the Korean population has been previously investigated in several studies, with similar findings (30–35). The results from the KNHANES 2007–2012 showed that the prevalence of elevated triglycerides was 17% lower in those who consumed a large amount of fruit than in those who did not (OR = 0.83, 95% CI = 0.71–0.98) (32). Pooled analyses also showed that milk and dairy product intake was associated with 18% decreased risks for both elevated triglycerides and low HDL cholesterol, with a pooled estimate (95% CI) of 0.82 (0.76–0.89) for both outcomes (11). In contrast, we did not find any associations between the prudent dietary pattern and dyslipidemia and its components, despite the high content of saturated fatty acids in dairy products (36, 37).

The recent guidelines for the management of dyslipidemia in Korea recommend the consumption of a healthy dietary pattern with a focus on whole grains (2/3-1 servings/meal), fruits (1-2 servings/day), vegetables (2.5-3 servings/meal), fish (including fatty fish), lean meat, eggs, and beans (38). Furthermore, comprehensive evidence from our previous meta-analysis of dietary intake and lipid profiles revealed protective effects of fruits (OR = 0.84, 95% CI = 0.73–0.96), eggs (OR = 0.84, 95% CI = 0.78–0.91), milk and dairy (OR = 0.82, 95% CI = 0.76–0.89), and coffee (OR = 0.84, 95% CI = 0.78–0.90) (11). In contrast, harmful effects were observed for the intake of sugar (OR = 1.24, 95% CI = 1.05–1.48), meat (OR = 1.14, 95% CI = 1.06–1.22), and sugar-sweetened beverages (OR = 1.20, 95% CI = 1.03–1.41) in a Korean population (11). However, in the current analysis of women only, we observed an increased prevalence of hyper-LDL cholesterol among those who consumed either a traditional or western diet. It was therefore recommended that women should limit their intake of both healthy and unhealthy foods and not to eat these foods excessively or too frequently in women.

When we stratified the study population according to menopausal status, the traditional dietary pattern was positively associated with the prevalence of hyper-LDL cholesterol in only postmenopausal women. This result suggested that among women with hyper-LDL cholesterol, conventional Korean foods were preferred in the postmenopausal group compared with the premenopausal group. Regarding the western dietary pattern, we also observed a significant association with both the prevalence of hypo-HDL cholesterol and hyper-LDL cholesterol in only postmenopausal subjects. This result may be explained by the increase in LDL cholesterol and the decrease in HDL cholesterol in women after menopause due to estrogen concentration changes; accordingly, the HDL cholesterol and LDL cholesterol levels in premenopausal women tended to be within the normal range (39–43). In particular, oral estrogen administration may accelerate the conversion of hepatic cholesterol to bile acids and enhance the LDL receptor expression (44). Estrogen using can also inhibit the activity of hepatic lipase and increase the production of apolipoprotein A (44). However, the pleiotropic mechanisms underlying the interactive associations of the traditional and western dietary patterns and menopausal status with dyslipidemia components remain unclear. Since receptors for estrogen and androgen are expressed in both visceral and subcutaneous adipocytes, alterations in sex hormones after menopause are associated with lipid metabolic disorders (5). Additionally, excessive visceral fat in postmenopausal women was related to an increased lipolysis rate of triacylglycerol into glycerol and free fatty acids, which resulted in a high concentration of fatty acid metabolites, such as heptanoate, octanoate, and pelargonate (5). Given the significantly lower intake of both plant and animal fat in postmenopausal women than in premenopausal women (Table 2), we hypothesized the induced effects of both the traditional diet on triglyceride reduction and the western diet on increasing total cholesterol in postmenopausal women.

The main strength of the current study is the inclusion of a large number of participants in the final analysis, which enabled an assessment of the effects of dietary patterns on dyslipidemia and its components in the total population as well as in premenopausal and postmenopausal women. The findings provide basic evidence for the prevention of dyslipidemia, which is a risk factor for cardiovascular disease. Compared with the use of the 24-h recall FFQ in other nationwide surveys, we obtained dietary behavior data via the validated FFQ, which provided data on the average food group consumption rates over the previous year (14, 45). Compared with the single food item approach, dietary patterns that are derived from data-driven approaches, such as factor analysis, address the interactions among food groups in a specific study population (46).

Despite its strengths, this study had several limitations. First, despite the large sample size, ~65% of the study population did not meet the eligibility criteria. Such a limited number of participants included in the final analysis may restrict the possibility of observing meaningful associations between dietary patterns and dyslipidemia. Second, the classification of the 24 food groupsmay be arbitrary. Additionally, the numbers of factors used for extraction and pattern labeling were subjective decisions (47). However, the main dietary patterns identified in our study were similar to those identified in previous studies using a statistical technique similar to factor analysis (17, 19). Third, although aging is known to be correlated with menopause, we were unable to consider the effects of both menopausal status and age in the analysis. Instead, we adjusted for age in the multivariable regression model and performed a stratified analysis to identify the effect modification of menopausal status. Fourth, although hormonal changes occurring in the body may affect lipid metabolism (12), we could not account for the effect of the follicular phase and the use of hormone therapy and oral contraceptives among study participants. Finally, the cross-sectional study design did not allow determination of the causal relationship between dietary patterns and dyslipidemia and its components.

In conclusion, the factor analysis identified three major dietary patterns in Korean women. There were significant associations between the traditional and western dietary patterns and hyper-LDL cholesterol in total and postmenopausal women, whereas non-significant associations were found for prudent dietary pattern. From the perspective of energy restriction, our findings recommend that women not to eat either traditional or western diet excessively or too frequently. Menopause may induce the effect of both the traditional diet on triglyceride reduction and the western diet on increasing total cholesterol.

## Data Availability Statement

The original contributions presented in the study are included in the article/supplementary material, further inquiries can be directed to the corresponding author/s.

## Ethics Statement

The studies involving human participants were reviewed and approved by National Cancer Center (number NCC2019-0204). The patients/participants provided their written informed consent to participate in this study.

## Author Contributions

JK, JL, TH, and SL: conceptualization, methodology, and writing—review and editing. JK: validation, supervision, and project administration. JL and TH: formal analysis. JK and JL: data curation. TH and SL: writing—original draft preparation. All authors contributed to the article and approved the submitted version.

## Funding

This study was supported by grant from the National Cancer Center Korea (2210990).

## Conflict of Interest

The authors declare that the research was conducted in the absence of any commercial or financial relationships that could be construed as a potential conflict of interest.

## Publisher's Note

All claims expressed in this article are solely those of the authors and do not necessarily represent those of their affiliated organizations, or those of the publisher, the editors and the reviewers. Any product that may be evaluated in this article, or claim that may be made by its manufacturer, is not guaranteed or endorsed by the publisher.

## Abbreviations

HDL: high-density lipoprotein
LDL: low-density lipoprotein
KNHANES: Korean National Health and Nutrition Examination Survey
NCC: National Cancer Center
FFQ: food frequency questionnaire
BMI: body mass index
OR: odds ratio
CI: confidence interval.
